# Editorial: Seed Dormancy, Germination, and Pre-harvest Sprouting

**DOI:** 10.3389/fpls.2018.01783

**Published:** 2018-11-30

**Authors:** Hiroyuki Nonogaki, Jose M. Barrero, Chengdao Li

**Affiliations:** ^1^Department of Horticulture, Oregon State University, Corvallis, OR, United States; ^2^Commonwealth Scientific and Industrial Research Organisation, Agriculture and Food, Canberra, ACT, Australia; ^3^Western Barley Genetics Alliance, Murdoch University, Murdoch, WA, Australia

**Keywords:** dormancy, seed germination, pre-harvest sprouting, late maturity alpha-amylase, functional genes, QTL

Seed germination is the first critical step of the plant life cycle and the foundation of agricultural production. In contrast, seed dormancy prevents seedling emergence in a wrong season or place in wild species. In cereal crops, the lack of dormancy results in pre-harvest sprouting (PHS) under particular conditions of humidity and temperature, which triggers reserve mobilization, mainly starch degradation by α-amylase (Bewley et al., 2013), and reduces grain quality. Annual losses from PHS are likely to approach $1 billion U.S. dollars worldwide (Black et al., 2006).

International Symposium on Pre-harvest Sprouting in Cereals gathered scientists investigating seed dormancy and PHS and staged an excellent opportunity for discussion between basic and applied researchers for the common goal-PHS prevention. This Research Topic: *Seed Dormancy, Germination, and Pre-harvest Sprouting* collected full papers and abstracts from the symposium and also other highly relevant papers submitted to the Journal. The collection also covers another grain quality defect that is often confounded with PHS: Late Maturity α-Amylase (LMA). This Research Topic serves as a reference book for PHS and LMA, in addition to the previous reviews for LMA and PHS (Mares and Mrva, 2014; Rodríguez et al., 2015).

Seed germination is promoted by GA. One way to prevent PHS is to understand the mechanisms of germination promotion by GA and block that pathway. Nelson et al. compared the transcriptomics of the *sleepy1-2* (*sly1-2*), a GA signaling mutant, and wild-type seeds and found dormancy-promoting stored transcripts. Mutations in these genes weaken seed dormancy and therefore their enhancement has potential to prevent PHS.

Another approach to strengthen seed dormancy is to reinforce ABA metabolism or signaling. Nonogaki and Nonogaki enhanced expression of *nine-cis-epoxycarotenoid dioxygenase* (*NCED*) and caused hyperdormancy. While the proof of concept was tested in Arabidopsis, the gene construct was created using a sorghum *NCED* and a wheat promoter. Therefore, the genetic tool is ready for direct translation into crops. *FUSCA3* (*FUS3*) enhances ABA biosynthesis in Arabidopsis (Gazzarrini et al., 2004). Sun et al. has found that *Triticum aestivum FUS3* (*TaFUS3*) interacts with TaSPA (STORAGE PROTEIN ACTIVATOR) (Albani et al., 1997). While the TaFUS3 analysis was not performed in the context of direct regulation of *NCED* expression, the interaction of TaFUS3 and TaSPA to transactivate gene expression advanced our understanding of the FUS3 function in ABA regulation.

ABI5 is involved in ABA signaling in seeds and regulated by nitric oxide (NO) (Nonogaki, 2017). Signorelli and Considine provided their perspectives about the action of reactive nitrogen species and the free radicals with an emphasis on post-translational modification. Understanding this pathway is also critical for PHS prevention and potentially more important for seed germination recovery from PHS-resistant crops because NO promotes germination.

The progresses in basic science are now ready to be translated into crops. However, the fundamental mechanism may have diversified among various crops. Therefore, species-specific case studies are also necessary. Das et al. performed metabolomic analysis using PHS-susceptible and -resistant wheat grains and presented significant differences in small molecule profiles. These profiles, together with proteomic and transcriptomic profiles, will identify crop-specific responses that might not be found in Arabidopsis. Benech-Arnold and Rodriguez shared their experience in PHS research in sorghum. While they also found a GA-ABA balance as the core mechanism of dormancy, GA deactivation, rather than ABA biosynthesis, turned out to be a critical factor for PHS resistance in sorghum. Their finding exemplifies the importance of comparative analyses between species. New QTL analyses in other crops offer a basis for cloning novel genes. Li et al. identified 43 QTL associated with low temperature seed germination of maize while Lee et al. identified 39 candidate SNPs for PHS by re-sequencing 21 representative accessions of rice. These new loci in maize and rice could potentially shed light on hidden pathways of seed dormancy and PHS. In the past, the rice QTL analysis for PHS identified *Seed dormancy 4* (*Sdr4*), which was not found from Arabidopsis (Sugimoto et al., 2010). Likewise, the analysis of the barley *Qsd1* identified the critical role of alanine aminotransferase in seed dormancy regulation through a yet-unknown pathway (Sato et al., 2016). Thus, more discoveries are anticipated from the crop QTL analyses.

This Research Topic also addresses inter- and intraspecies differences in dormancy and PHS mechanisms by using wild, local or synthetic species. Nakamura et al. crossed the wild barley to a malting barley and highlighted the importance of maternal effects. Dale et al. crossed *Aegilops tauschii* with a hexaploid PHS-susceptible white-grain wheat and generated a synthetic octaploid wheat. By using the advanced backcross population, they identified dormancy-associated QTLs. In addition to wild or synthetic species, it is also important to use varieties of local origin for addressing geographical and climate differences of PHS responses in a global scale. Sydenham and Barnard conducted QTL analysis of the South African wheat cultivars while Zhou et al. performed genome-wide association study of the large germplasm collection of Chinese landraces.

The ultimate goal of PHS research is to secure grain quality. Zhang and Li employed genomic approaches to compare α-amylase genes in cereals, including promoters. The information is a great resource to understand the “behavior” of the starch-degrading enzyme and regulate its expression for grain quality. Unusual α-amylase activity in late maturing wheat grains, so-called LMA, is a genetic defect and considered a negative trait for grain quality. However, Newberry et al. are now raising question about the traditional view about LMA. They observed little correlation between LMA and the end product functionality and concluded that LMA has limited impact on bread baking. Martinez et al. even demonstrated inconsistency between some falling number and PHS traits. These new discoveries could be a game changer in breeding programs.

Finally, another important aspect of PHS studies is the synergy between basic and applied science. As discussed for *Sdr4* and *Qsd1*, crop research revealed the presence of novel pathways of seed dormancy, which was not known in model species. The *mitogen-activated protein kinase kinase 3*(*TaMKK3*) is emerging as a novel seed dormancy regulator in crops (Nakamura et al., 2016; Torada et al., 2016). Shorinola et al. analyzed a causal relation between TaMKK3 and PHS in the global germplasm and confirmed its significance for seed dormancy. TaMKK3 also offers a new target pathway to be investigated in basic seed dormancy research using Arabidopsis. Thus, PHS studies are advancing both basic seed biology and applied crop science.

## Author contributions

All authors listed have made a substantial, direct and intellectual contribution to the work, and approved it for publication.

### Conflict of interest statement

The authors declare that the research was conducted in the absence of any commercial or financial relationships that could be construed as a potential conflict of interest.
